# Airway ciliary dysfunction and respiratory symptoms in patients with transposition of the great arteries

**DOI:** 10.1371/journal.pone.0191605

**Published:** 2018-02-14

**Authors:** Maliha Zahid, Abha Bais, Xin Tian, William Devine, Dong Ming Lee, Cyrus Yau, Daniel Sonnenberg, Lee Beerman, Omar Khalifa, Cecilia W. Lo

**Affiliations:** 1 Dept. of Developmental Biology, University of Pittsburgh School of Medicine, Pittsburgh, Pennsylvania, United States of America; 2 Office of Biostatistics Research, National Heart Lung Blood Institute, Bethesda, Maryland, United States of America; 3 Dept. of Pathology, University of Pittsburgh School of Medicine, Pittsburgh, Pennsylvania, United States of America; 4 Division of Pediatric Cardiology, Department of Pediatrics, University of Pittsburgh School of Medicine, Pittsburgh, Pennsylvania, United States of America; Vanderbilt University Medical Center, UNITED STATES

## Abstract

**Background:**

Our prior work on congenital heart disease (CHD) with heterotaxy, a birth defect involving randomized left-right patterning, has shown an association of a high prevalence of airway ciliary dysfunction (CD; 18/43 or 42%) with increased respiratory symptoms. Furthermore, heterotaxy patients with ciliary dysfunction were shown to have more postsurgical pulmonary morbidities. These findings are likely a reflection of the common role of motile cilia in both airway clearance and left-right patterning. As CHD comprising transposition of the great arteries (TGA) is commonly thought to involve disturbance of left-right patterning, especially L-TGA with left-right ventricular inversion, we hypothesize CHD patients with transposition of great arteries (TGA) may have high prevalence of airway CD with increased respiratory symptoms.

**Methods and results:**

We recruited 75 CHD patients with isolated TGA, 28% L and 72% D-TGA. Patients were assessed using two tests typically used for evaluating airway ciliary dysfunction in patients with primary ciliary dyskinesia (PCD), a recessive sinopulmonary disease caused by respiratory ciliary dysfunction. This entailed the measurement of nasal nitric oxide (nNO), which is typically low with PCD. We also obtained nasal scrapes and conducted videomicroscopy to assess respiratory ciliary motion (CM). We observed low nNO in 29% of the patients, and abnormal CM in 57%, with 22% showing both low nNO and abnormal CM. No difference was observed for the prevalence of either low nNO or abnormal ciliary motion between patients with D vs. L-TGA. Respiratory symptoms were increased with abnormal CM, but not low nNO. Sequencing analysis showed no compound heterozygous or homozygous mutations in 39 genes known to cause PCD, nor in CFTR, gene causing cystic fibrosis. As both are recessive disorders, these results indicate TGA patients with ciliary dysfunction do not have PCD or cystic fibrosis (which can cause low nNO or abnormal ciliary motion).

**Conclusions:**

TGA patients have high prevalence of abnormal CM and low nNO, but ciliary dysfunction was not correlated with TGA type. Differing from PCD, respiratory symptoms were increased with abnormal CM, but not low nNO. Together with the negative findings from exome sequencing analysis, this would suggest TGA patients with ciliary dysfunction do not have PCD but nevertheless may suffer from milder airway clearance deficiency. Further studies are needed to investigate whether such ciliary dysfunction is associated with increased postsurgical complications as previously observed in CHD patients with heterotaxy.

## Introduction

Congenital heart disease (CHD) is the most common congenital disorder with prevalence of 6 to 21 per 1000 live births [1, 2]. Some of the most complex CHD is associated with laterality defects, a birth defect involving randomization in left-right patterning. Patients with CHD and laterality defects have a high prevalence (42%) of airway ciliary dysfunction (CD) [3], indicated by the finding of low nasal nitric oxide (nNO) and abnormal ciliary motion (CM), findings reminiscent of patients with primary ciliary dyskinesia (PCD), a sinopulmonary disease associated with dyskinetic/immotile cilia in the airway. We previously showed patients with CD exhibited more respiratory symptoms and those undergoing cardiac surgery had more postsurgical respiratory complications [3, 4]. These findings may reflect the common requirement for motile cilia in embryonic left-right patterning and airway clearance. This is supported by studies showing mutations causing PCD are associated with a high incidence of laterality defects and complex CHD, findings observed in both mouse models and human clinical studies [5].

Transposition of the great arteries (TGA) is a complex CHD that is thought to involve a defect in left-right patterning. In D-TGA, ventriculo-arterial discordance occurs with anterior aorta positioning and insertion into the right ventricle, while in L-TGA, there is additional ventricular inversion with the morphological right ventricle positioned on the body’s left, also known as congenitally corrected TGA. Hence, TGA, especially L-TGA, is suggested to have a common developmental etiology with laterality defects [6]. Consistent with this, mutations in genes known to regulate left-right patterning have been observed in TGA patients [6–10]. We note mice with mutations in *Dnai1* or *Dnah5*, genes known to cause PCD, can have complex CHD associated with laterality defects including either D- or L-TGA, [11–13]. Based on these findings, we hypothesize TGA patients may have a high prevalence of respiratory CD with more respiratory symptoms and disease. To test this hypothesis, we recruited patients with isolated D and L-TGA without any other visceral organ laterality defects and assessed for respiratory CD and respiratory symptoms/disease. Our studies showed patients with transposition of great arteries (TGA) have high prevalence of airway CD with more respiratory symptoms, but this is likely distinct from classic PCD.

## Methods

### Patient recruitment

TGA patients were recruited from Children’s Hospital of Pittsburgh with University of Pittsburgh’s Institutional Review Board approved protocol and in accordance with approved guidelines. Informed consent was obtained from all patients, or in case of minors, from their parents/guardians. All TGA patients with concomitant laterality defects, as well as atrial or bronchial isomerism were excluded after systematic review (by WD, CY) of surgical reports, and all imaging data available on these patients. This included findings obtained from echocardiography, chest X-rays, abdominal X-rays, CT scan of the chest and/or abdomen, and MRI performed for pre-operative planning or for a clinical indication. Respiratory symptoms were obtained using detailed questionnaire and medical chart review. Respiratory symptoms were tracked using 12 respiratory symptoms grouped into four categories: all respiratory symptoms (ARS), PCD symptoms (otitis media, sinusitis, chronic cough, chronic sputum production, neonatal respiratory distress, pneumonia and bronchiectasis), upper respiratory symptoms (URT; otitis media, sinusitis, nasal polyps, chronic nasal congestion, allergic rhinitis), and lower respiratory symptoms (LRT; bronchitis, chronic cough, neonatal pneumonia/respiratory distress, bronchiectasis, pneumonia/chest infections, asthma, recurrent wheezing).

### Nasal tissue sampling, reciliation and ciliary motion analysis

Nasal tissue was obtained by curettage of the inferior nasal turbinate using a Rhino-Probe (Arlington Scientific, Springville, UT) and processed for video-microscopy [3]. Ciliary beat frequency and CM analysis was carried out by a panel of investigators (M.Z., R.F., C.W.L.) blinded to the clinical status, nNO levels and phenotype of the patient. The nasal epithelial tissue was cultured for reciliation, to rule out secondary CM defects, using previously described methods (Supplemental Methods) [3].

### Nasal nitric oxide measurement

Nasal nitric oxide measurements were made using the CLD88 SP (Ecophysics AG) NO analyzer with protocols recommended by the American Thoracic Society/European Respiratory Society [14], with velum closure technique for participants >6yrs, and tidal breath sampling for participants <6yrs (Supplemental Methods) [15]. Consistent with the American Thoracic Society’s recommendations for nNO levels [14], we defined normal nNO with a cut-off value of >100nl/min for those between 1-6yrs old and >200nl/min for >6yrs of age. For patients <1 yr of age, we utilized cut-off values obtained from normal infants using the tidal breathing method that we have published [16].

### Whole exome DNA sequencing

DNA was extracted from blood for 65 of the 75 patients and whole-exome sequencing (WES) performed using Agilent SureSelect All Exon Kit V4 and Illumina High-seq2000 sequencer. An average coverage of ~100x was achieved. Exome sequencing data was processed with a pipeline based on BWA (v0.5.9), Picard tools and GATK HaplotypeCaller and annotated with Variant Effect Predictor (v89) with a single consequence retrieved for each variant. Variants were subsetted to the ExAC calling intervals and filtering performed to focus on rare coding variants. All known PCD genes [17] (Supplemental Spreadsheet) except *HYDIN* (excluded due to technical artifact with sequence hypervariability) were examined for novel or rare coding variants (nonsynonymous, frameshift, splicing), defined as those with an ExAC adjusted allele frequency < = 0.01 and a CADD PHRED[18] score of at least 10. Pathogenicity of these variants was assessed by PolyPhen-2 [19] and SIFT [20]. ExAC VCF file [21] was re-annotated and processed similarly. Human Gene Mutation Database (HGMD) was searched for all known pathogenic PCD gene mutations [22].

### Statistical analysis

Chi-square test and the Mann-Whitney test were used to analyze CM, nNO levels and individual respiratory symptoms for normally and non-normally distributed data respectively. Combination of respiratory symptoms were used as the dependent variable and association with age, CM abnormalities, nNO levels and TGA-type examined by univariable and multivariable linear regression. A two-sided p-value of <0.05 was considered significant. Analyses were performed with Stata 12.1 (Stata Corporation, College Station, TX).

## Results

**Fig 1:**
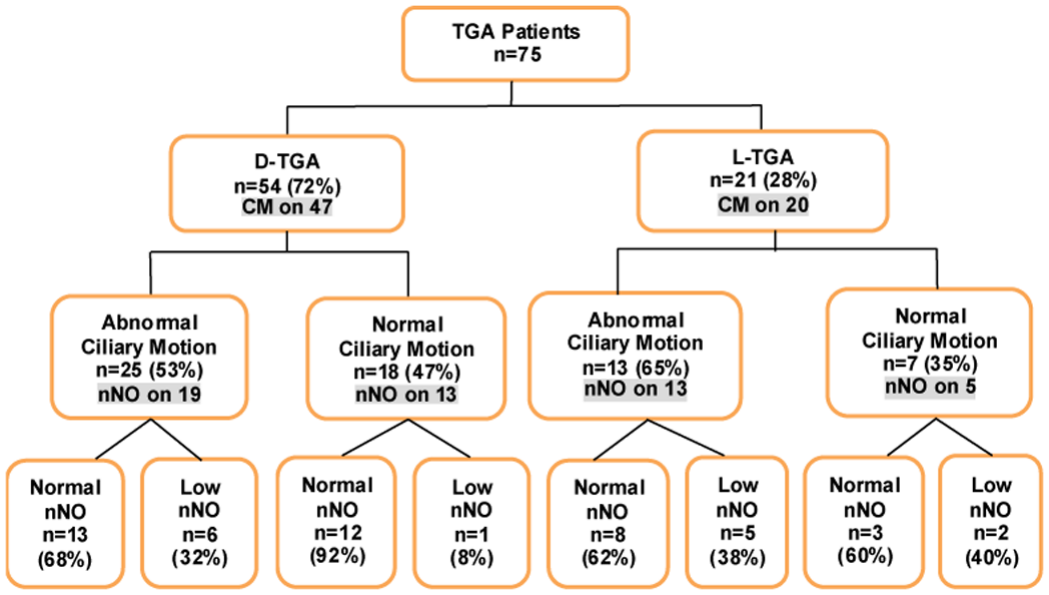
Summary of TGA patients assessed for both ciliary motion abnormalities and low nNO levels. Shown in the flowchart are 75 TGA patients with ciliary motion available in 67 and nNO values available on 50 patients, broken down into normal and abnormal within each type of TGA.

We recruited 75 patients with isolated TGA without concomitant visceral organ laterality defects (Fig 1; S1 Fig). Patient cohort was mostly Caucasian, 68% male (S1 Table), with 54 being D-TGA and 21 L-TGA (Fig 1; S2 Table). Nasal scrapes were conducted for ciliary motion analysis on 67 of the 75 patients, and nNO was obtained in 63 patients (Fig 1). Patient respiratory symptoms were obtained from each patient, and stratified into all respiratory symptoms (ARDS), upper and lower respiratory symptoms (URT, LRT) and PCD symptoms.

### Analysis of ciliary motion

Analysis of 67 TGA patients with CM data available showed 29 (43%) with normal CM (S1 Movie), and 38 (57%) with abnormal CM, ranging from stiff/dyskinetic ciliary motion (S2 Movie), incomplete (decreased amplitude; S3 Movie) or wavy stroke (S4 Movie), or asynchronous motion (S5 Movie). The prevalence of CM was not significantly different in L- (13/20; 65%) versus D-TGA (25/47; 53%) (p = 0.372). Analysis of ciliary beat frequency showed no difference between D (6.1±1.4) versus L-TGA (6.5±2.0) (S1 Table) or amongst all TGA patients (6.2±1.6) compared to control subjects (6.0 ±1.7). To rule out abnormal CM due to secondary causes, CM was analyzed again after reciliation in vitro (Supplemental Methods). In 9 patients with normal CM, normal CM was replicated after reciliation (S6A and S6B Movie). In 8 patients with abnormal CM, 6 (75%) were confirmed as abnormal, while 2 showed normal CM (S7A and S7B Movie), yielding a correlation coefficient of 0.78 (p-value<0.001). In discrepant cases, the final CM phenotype was based on the reciliation results.

### Nasal nitric oxide assessments

Nasal NO measurements were obtained for 63 TGA patients, and were compared to nNO values from 113 healthy controls and 41 known PCD subjects (S3 Table; Table 1). Using normative values established for nNO in subjects <1 year old [23], we showed TGA patients <1yr (n = 19) had nNO values (9.7±5.6 nl/min) considerably lower than healthy controls (68±23.4 nl/min) and similar to those seen in PCD patients (7.8±5.2) (Table 1). No analysis was carried out for the 1–6 yrs group, given this comprised only 2 patients. For TGA patients >6yrs (n = 54), the mean nNO was 246±98 nl/min, significantly lower than healthy controls (316.3±90 nl/min, p = 0.0026; Table 1), but much higher than levels seen in PCD patients (16.5±10.5, p<0.0001; Table 1). L-TGA patients had somewhat lower nNO (223±102nl/min) as compared to D-TGA (263±93nl/min) that bordered on being significant (p = 0.0536; Table 1).

**Table 1:** Nasal NO measurements in TGA patients.

	nNO (nl/min)	Normal CM	Abnormal CM	Low nNO/ Nml CM	Low nNO/ Abn CM	Healthy Control	PCD Patients
D+L-TGA <1- yr (n = 19)	9.7±5.6	11.5±6.3 (n = 9) p = ns^†^ p = 0.0004^‡^	7.7±3.9 (n = 6) p = ns^†^ p = 0.0012^‡^	-	-	68.8±23.4 (n = 8)	7.8±5.2 (n = 6)
		p = 0.0593					
D+L-TGA 1–6 yr (n = 2)	40.0±37.1	-	66.2 (n = 1)	-	66.2 (n = 1)	123.3±59.6 (n = 82)	19.6±13.7 (n = 17)
D+L-TGA >6 yr (n = 54)	246±98 p<0.0001^†^ p = 0.0026^‡^	255±61 (n = 14) p<0.0001^†^ p = 0.0412^‡^	239±111 (n = 28) p<0.0001^†^ p = 0.0031^‡^	180±12 (n = 3) p = 0.0066^†^ p = 0.0199^‡^	141±44 (n = 10) p<0.0001^†^ p<0.0001^‡^	316.3±90 (n = 26)	16.5±10.5 (n = 18)
		p = ns		p = 0.0630			
D-TGA* >6 yrs (n = 26)	263±93 p<0.0001^†^ p = 0.0558^‡^	271±64 (n = 9) p<0.0001^†^ p = ns^‡^	247±107 (n = 15) p<0.0001^†^ p = 0.0397^‡^	176.7 (n = 1)	137.5±53.7 (n = 5) P = 0.0008^†^ P = 0.0009^‡^	316.3±90.0 (n = 26)	16.5±10.5 (n = 18)
		p = ns		-			
L-TGA* >6 yrs (n = 19)	223±102 p<0.0001^†^ p = 0.0007^‡^	227±48 (n = 5) p = 0.0008^†^ p = 0.0338^‡^	230±118 (n = 13) p<0.0001^†^ p = 0.0046^‡^	182.1 (n = 2)	144.4±37.2 (n = 5) P = 0.0008^†^ P = 0.0007^‡^	316.3±90.0 (n = 26)	16.5±10.5 (n = 18)
		p = ns		-			

^†^Mann Whitney U test comparison with PCD.

^‡^ Mann Whitney U test comparison with Controls.

*Comparison between D vs. L-TGA yielded p = 0.0536

### Combined analysis of nNO and ciliary motion

For 50 of the TGA patients >1yr age, we obtained both nNO and CM data (Table 2). Stratifying the TGA patients based on CM finding showed those with abnormal CM had somewhat lower nNO, but the difference was not significant, either with comparison among all TGA patients, or within D or L-TGA patient subgroups (Table 1). We further stratified these patients into those with low nNO using ATS guideline of <100 for 1–6 yrs old and <200 nl/min for those >6 yrs old [14] as in our previous study [16]. The nNO levels in patients with low nNO and abnormal CM were lower than those with low nNO and normal CM, with a trend towards statistical significance (p = 0.063; Table 1). Further stratification based on L vs. D-TGA phenotype was not informative given the very small sample size (Table 1). Overall, 11 (22%) patients had abnormal CM/low nNO and 15 (30%) had normal CM/normal nNO (Table 2). We also observed 3 (6%) patients with low nNO had normal CM, and 21 (42%) with abnormal CM had normal nNO. There was no significant difference in the prevalence of patients with abnormal CM/low nNO by TGA-type (Table 2).

**Table 2:** Abnormal CM and low NO prevalence with D vs. L-TGA.

	D-TGA	L-TGA	p-value
Total assessed for NO and CM	32 (64%)	18 (36%)	
Abn NO + Abn CM	6 (19%)	5 (28%)	p = 0.494
Nml NO + Nml CM	12 (38%)	3 (17%)	p = 0.199
Abn NO + Nml CM	1 (3%)	2 (11%)	p = 0.291
Nmll NO + Abn CM	13 (41%)	8 (44%)	p = 1.000

### Respiratory symptoms associated with abnormal ciliary motion

Abnormal CM was significantly associated with ARS (p = 0.004), PCD symptoms (p = 0.007), LRT symptoms (p = 0.018) and URT symptoms (p = 0.018) (Table 3, S4 Table). In contrast, low nNO (<200 nl/min) was not significantly correlated with any of these respiratory symptom categories, although a trend towards significance was observed for increase in ARS (p = 0.078; Table 3) and PCD (p = 0.071; Table 3) symptoms. No difference between respiratory symptoms was observed in any categories when comparing between TGA-types.

**Table 3:** Increased respiratory symptoms in TGA patients >6 yrs of age with abnormal ciliary motion and low nNO.

	All Symptoms		PCD Symptoms*		LRT Symptoms^†^		URT Symptoms^‡^	
Ciliary Motion (n = 48)	Normal CM	Abn. CM	Normal CM	Abn. CM	Normal CM	Abn. CM	Normal CM	Abn. CM
Mean No. Symptoms ± SD	0.59 ± 1.2	2.29 ± 2.3	0.29 ± 0.6	1.26 ± 1.3	0.18 ± 0.5	1.0 ± 1.5	0.41 ± 0.8	1.29 ± 1.4
	p = 0.004		p = 0.007		p = 0.018		p = 0.018	
Percent with Symptoms	9 (45%)	22 (79%)	12 (48%)	19 (83%)	17 (53%)	14 (88%)	12 (50%)	19 (79%)
	p = 0.017		p = 0.012		p = 0.019		p = 0.035	
nNO (n = 43)	Normal nNO	Low nNO	Normal nNO	Low nNO	Normal nNO	Low nNO	Normal nNO	Low nNO
Mean No.Symptoms ± SD	1.4 ± 1.9	2.4 ± 1.9	0.7 ± 1.1	1.4 ± 1.3	0.7 ± 1.3	0.8 ± 1.1	0.7 ± 1.0	1.5 ± 1.6
	p = 0.078		p = 0.071		p = ns		p = ns	
Percent with Symptoms	15 (50%)	10 (77%)	13 (43%)	9 (69%)	9 (30%)	6 (46%)	14 (47%)	8 (62%)
	p = 0.100		p = ns		p = ns		p = ns	
Cilia Motion and nNO (n = 41)	All Other Groups	Abn. CM Low nNO	All Other Groups	Abn. CM Low nNO	All Other Groups	Abn. CM Low nNO	All Other Groups	Abn. CM Low nNO
Mean No.Symptoms ± SD	1.3 ± 2.1	3.1 ± 1.6	0.7 ± 1.1	1.8 ± 1.1	0.6 ± 1.3	1.1 ± 1.2	0.7 ± 1.1	2.0 ± 1.5
	p = 0.001		p = 0.002		p = 0.069		p = 0.007	
Percent with Symptoms	15 (47%)	10 (100%)	14 (37%)	9 (90%)	10 (26%)	6 (60%)	16 (42%)	8 (80%)
	p = 0.003		p = 0.003		p = 0.044		p = 0.003	
TGA-Type (n = 51)	D-TGA	L-TGA	D-TGA	L-TGA	D-TGA	L-TGA	D-TGA	L-TGA
Mean No.Symptoms ± SD	1.8 ± 2.1	1.6 ± 2.1	1 ± 1.2	0.8 ± 1.2	0.8 ± 1.4	0.6 ± 0.9	0.9 ± 1.2	1.1 ± 1.3
	p = ns		p = ns		p = ns		p = ns	
Percent with Symptoms	21 (64%)	9 (50%)	17 (52%)	8 (44%)	12 (36%)	6 (33%)	17 (52%)	9 (50%)
	p = ns		p = ns		p = ns		p = ns	

*PCD Symptoms: Otitis Media, Pneumonia/Chest Infections, Neonatal Pneumonia/Respiratory Distress Syndrome, Chronic Cough, Bronchiectasis, Sinusitis.

^†^LRT Symptoms (7): Lower Respiratory Tract Symptoms (Bronchitis, Chronic Cough, Neonatal Pneumonia/Respiratory Distress Syndrome, Bronchiectasis, Pneumonia/Chest Infections, Asthma, Recurrent Wheezing).

^‡^URT Symptoms (5): Upper Respiratory Tract Symptoms (Otitis Media, Sinusitis, Nasal Polyps, Chronic Nasal Congestion, Allergic Rhinitis)

Univariable regression analyses showed abnormal CM was significantly associated with increase in all 4 respiratory symptom categories, while low nNO was only significantly associated with URT symptoms (p = 0.046; Table 4) with a trend towards increased PCD symptoms (p = 0.092; Table 4). In contrast, age or TGA-type had no significant correlation with any of the respiratory symptom categories. Placing both low nNO and CM in a multivariable regression analysis (Table 5) showed significant association of abnormal CM with ARS (p = 0.022), PCD (p = 0.007) and URT (p = 0.019) symptoms, but no significant association was observed between low nNO and any respiratory symptom categories after correcting for abnormal CM (Table 5).

**Table 4:** Univariable regression analysis of respiratory symptoms in TGA patients>6 Yr.

Independent Variable	All Symptoms		PCD Symptoms*		LRT Symptoms^†^		URT Symptoms^‡^	
Covariate	β	*P-value*	β	*P-value*	β	*P-value*	β	*P-value*
Age at Enrollment	0.010	0.711	-0.001	0.967	-0.007	0.650	0.017	0.279
Abnormal vs. Normal CM	1.702	0.006	0.964	0.007	0.824	0.030	0.879	0.019
Low vs. Normal nNO	0.985	0.134	0.651	0.092	0.179	0.668	0.805	0.046
TGA Type: D vs. L	-0.146	0.812	-0.136	0.702	-0.263	0.479	0.116	0.753

**Table 5:** Multivariable regression analysis of respiratory symptoms in TGA patients>6 Yr.

Independent Variable	All Symptoms		PCD Symptoms*		LRT Symptoms^†^		URT Symptoms^‡^	
Covariate	β	*P-value*	β	*P-value*	β	*P-value*	β	*P-value*
Abnormal vs. Normal CM	1.497	0.022	0.876	0.023	0.683	0.111	0.814	0.044
Low vs.Normal nNO	0.801	0.208	0.524	0.166	0.117	0.781	0.686	0.087

*PCD Symptoms (6 of the 12 symptoms): Otitis Media, Pneumonia/Chest Infections, Neonatal Pneumonia/Respiratory Distress Syndrome, Chronic Cough, Bronchiectasis, Sinusitis.

^†^ LRT Symptoms (7): Lower Respiratory Symptoms consisting of Bronchitis, Chronic Cough, Neonatal Pneumonia/Respiratory Distress Syndrome, Bronchiectasis, Pneumonia/Chest Infections, Asthma, Recurrent Wheezing.

^‡^ URT Symptoms (5): Upper Respiratory Symptoms consisting of Otitis Media, Sinusitis, Nasal Polyps, Chronic Nasal Congestion, Allergic Rhinitis.

### Sequencing analysis for mutations in PCD genes

We conducted whole exome sequencing analysis for 65 of the TGA patients (S2 Table), focusing our analysis on the 39 genes known to cause PCD[17]. We recovered 90 rare coding variants in 28 PCD genes, with 47 of the variants predicted to be damaging (Table 6), but none are curated as disease causing in the Human Gene Mutation Database (HGMD). There were 0.72 damaging variants per TGA patient, which is significantly greater than the 0.11 variants per subject observed in similar analysis of exome sequencing data from 60,706 subjects in the ExAC cohort (p<0.01) (Table 5). Overall, the mutation load was significantly higher, and included more damaging mutations in the TGA patients as compared to the ExAC cohort (Table 6). There was no difference in prevalence of damaging PCD mutations between D vs. L TGA patients, TGA patients with abnormal vs. normal ciliary motion, or low vs. normal nNO (Table 6). We also interrogated for mutations in *CFTR*, gene known to cause cystic fibrosis (CF), since respiratory ciliary dysfunction can occur secondary to CF. This analysis yielded only four heterozygous mutations that are curated as either disease causing or possibly disease causing in the HGMD, excluding CF which is a recessive disorder (Supplementary Excel Spreadsheet).

**Table 6:** Analysis of incidence of PCD gene mutations in TGA cohort.

Variants	ExAC N = 60,706	TGA N = 65	D-TGA N = 46	L-TGA N = 19	Abn CM N = 36	Norm CM N = 25	Low nNO N = 19	Norm nNO N = 35
All Coding Variants	3,019,601	20,461	14,862	5,599	10,585	7,779	6,438	10,694
Per subject	49.74	314.78	323.09	294.68	294.03	311.16	338.84	305.54
	p<0.01*		p<0.01		p<0.01		p<0.01	
All Damaging Coding Variants	1,545,281	9,440	6,833	2,607	4,852	3,671	2,979	4,886
Per subject	25.46	145.23	148.54	137.21	134.78	146.84	156.79	139.60
	p<0.01*		p<0.01		p<0.01		p<0.01	
PCD Coding Variants	11,567	90	61	29	48	35	29	52
Per subject	0.19	1.38	1.33	1.53	1.33	1.40	1.53	1.49
	p<0.01*		p<0.01		p<0.01		p<0.01	
PCD Damaging Coding Variants	6391	47	32	15	22	20	15	27
Per subject	0.11	0.72	0.70	0.79	0.61	0.80	0.79	0.77
	p<0.01*		p = NS		p = NS		p = NS	

*Comparison of TGA cohort against the ExAC cohort was made using the Z-statistic.

## Discussion

We observed a high prevalence of abnormal CM (38/76; 57%) and/or low nNO in isolated TGA patients, with 22% exhibiting both abnormal CM and low nNO. Abnormal CM was associated with increased respiratory symptoms. This finding is unexpected, since these patients do no exhibit heterotaxy, and yet yielded results similar to our previous study of heterotaxy patients [3]. In the latter study, we observed 4 of 15 (26%) heterotaxy patients with TGA exhibited both low nNO and abnormal CM. Together, these findings suggest TGA pathogenesis with or without laterality defects may have common involvement of motile cilia defects. We noted a trend towards higher incidence of abnormal CM and low nNO in L-TGA patients. This suggests the possibility that some cases of L-TGA may involve laterality disturbance despite the absence of obvious visceral organ situs anomalies.

Some TGA patients with abnormal CM showed normal nNO (42%), while low nNO (6%) were observed with normal CM. However, nNO levels did not differ between TGA patients with normal vs. abnormal CM. We note the overall prevalence of abnormal CM may be somewhat overestimated, as reciliation analysis showed the original CM phenotype was not replicated in 2 of 17 patients. This potentially could account for some of the patients with abnormal CM that have normal nNO.

Discordant CM/nNO findings also have been noted in our previous study of heterotaxy patients and in a more recent study of patients with CHD of a broad spectrum [3, 16]. A high prevalence of CD was also observed in the latter study, suggesting a broader role for respiratory CD in CHD pathogenesis [16]. While PCD patients usually have abnormal CM in conjunction with low nNO, in fact not all PCD patients have low nNO [24–26]. However as none of our patients had biallelic mutations in any of the known PCD genes, our patient population is likely distinct from PCD. Cystic fibrosis, which also can cause low nNO, was excluded in 65 of our TGA patients by WES. We also note none of our patients had clinical manifestations of cystic fibrosis.

We found TGA patients with abnormal CM had increased respiratory symptoms whether stratified as all respiratory symptoms (ARS), PCD symptoms, or lower (LRT) or upper (URT) symptoms. Analysis in a univariable model showed patients with low nNO also had increased URT symptoms, with PCD symptoms showing a trend towards significance. Correcting for low nNO in a multivariate model, abnormal CM remained significantly associated with ARS, PCD and URT symptoms, but low nNO was no longer significant for any of the respiratory symptom groupings. This negative finding may be due to the small sample size, as in a larger study of patients with CHD of a broad spectrum, we noted abnormal CM and low nNO were each significantly associated with ARS, PCD and LRT symptoms [16]. However, it is worth noting that in another study of patients with CHD of a broad spectrum without heterotaxy, we found those with abnormal ciliary motion, but not low nNO, had increased postsurgical respiratory complications [27].

TGA patients with low nNO had nNO values that were mostly higher than those observed with PCD, similar to our previous analysis of CHD patients with or without heterotaxy [3, 16]. These findings further suggest CHD patients with low nNO and abnormal CM are largely distinct from PCD. Consistent with this, neonatal respiratory distress syndrome often seen with PCD was reported in only 6 of the 75 TGA patients– 4 with abnormal CM. Furthermore, the results of our WES analysis also indicated that the TGA patients with ciliary dysfunction are not likely to have PCD. Comparison with the ExAC cohort suggested there may be an increase in mutation load overall in the TGA patients, including more rare coding variants in PCD genes in the TGA patients. However, the prevalence of PCD mutation was not significantly elevated in patients with abnormal CM or low nNO, further indicating that TGA patients with ciliary dysfunction do not have classic PCD.

There are several limitations to our study. This was a relatively small patient cohort which may account for the failure to observe a significant correlation between low nNO and respiratory symptoms. In particular, the much lower number of L-TGA patients may have limited our ability to detect differences between patients of the D vs.L-TGA subtypes. Another limitation is the possible contribution of secondary dyskinesia to abnormal CM, which was partially addressed with CM analysis after reciliation in a subset of patients.

In conclusion, our study corroborated the hypothesis that TGA patients without concomitant laterality defects have a high prevalence of ciliary motion abnormalities. The prevalence of ciliary dysfunction showed no correlation with TGA type, indicating L vs D-TGA do not differ in regards to the involvement of left-right patterning disturbance. While our findings showed TGA patients with CD are distinct from PCD patients, they nevertheless exhibited increased respiratory morbidity suggestive of airway clearance deficiency. Whether this might be associated with increased postsurgical complications will need to investigated (4,27). Overall, these findings support the notion that TGA patients may benefit from preoperative screening for CM abnormalities, and prophylactic respiratory therapies to help improve postsurgical outcomes.

## Supporting information

S1 Table Cilia motion and nNO in TGA patients. (DOCX)

S2 Table Detailed cardiovascular anatomy in TGA patients. (DOCX)

S3 Table Nasal NO values by age groups in PCD and healthy controls. (DOCX)

S4 Table Respiratory symptoms in patients >6 yrs of age. (DOCX)

S1 Fig Cilia motion analysis and nNO measurement in the TGA cohort. (DOCX)

S1 Movie Video-microscopy of nasal epithelia from a healthy control subject showing normal, synchronous, metachronal ciliary motion. (MOV)

S2 Movie Video-microscopy of nasal epithelia from TGA patient 7286 showing stiff, dyskinetic and in some places immotile cilia. (MOV)

S3 Movie Video-microscopy of nasal epithelia from a TGA patient 7235 showing ciliary motion with incomplete stroke. (MOV)

S4 Movie Video-microscopy of nasal epithelia from TGA patient 7096 showing wavy stroke. (MOV)

S5 Movie Video-microscopy of nasal epithelia from TGA patient 7152 showing asynchronous ciliary beating. (MOV)

S6 Movie A, B: Video-microscopy of nasal epithelia from TGA patient 7129 showing normal ciliary motion on initial nasal scrape (A) and upon reciliation (B).
(MOV)

S7 Movie A, B: Video-microscopy of nasal epithelia from TGA patient 7273 showing abnormal ciliary motion (asynchronous, incomplete stroke) on initial nasal scrape (A) and upon reciliation (B).
(MOV)

S1 Methods Supplemental detailed methods can be found here. (DOC)

S1 File SupplementalSpreadsheet_PCDMutations. This is a list of all the PCD mutations identified in the sequenced TGA patients.
(XLSX)

## References

[1] Reller MD, Strickland MJ, Riehle-Colarusso T, Mahle WT, Correa A. Prevalence of congenital heart defects in metropolitan Atlanta, 1998–2005. The Journal of pediatrics. 2008;153(6):807–13. Epub 2008/07/29. doi: 10.1016/j.jpeds.2008.05.059 . 18657826 10.1016/j.jpeds.2008.05.059 PMC2613036

[2] Wren C, Irving CA, Griffiths JA, O′Sullivan JJ, Chaudhari MP, Haynes SR, et al Mortality in infants with cardiovascular malformations. European journal of pediatrics. 2012;171(2):281–7. Epub 2011/07/13. doi: 10.1007/s00431-011-1525-3 . 21748291 10.1007/s00431-011-1525-3

[3] Nakhleh N, Francis R, Giese RA, Tian X, Li Y, Zariwala MA, et al High prevalence of respiratory ciliary dysfunction in congenital heart disease patients with heterotaxy. Circulation. 2012;125(18):2232–42. Epub 2012/04/14. doi: 10.1161/CIRCULATIONAHA.111.079780 . 22499950 10.1161/CIRCULATIONAHA.111.079780 PMC3770728

[4] Harden B, Tian X, Giese R, Nakhleh N, Kureshi S, Francis R, et al Increased postoperative respiratory complications in heterotaxy congenital heart disease patients with respiratory ciliary dysfunction. J Thorac Cardiovasc Surg. 2014;147(4):1291–8 e2. doi: 10.1016/j.jtcvs.2013.06.018 . 23886032 10.1016/j.jtcvs.2013.06.018

[5] Kennedy MP, Omran H, Leigh MW, Dell S, Morgan L, Molina PL, et al Congenital heart disease and other heterotaxic defects in a large cohort of patients with primary ciliary dyskinesia. Circulation. 2007;115(22):2814–21. Epub 2007/05/23. doi: 10.1161/CIRCULATIONAHA.106.649038 . 17515466 10.1161/CIRCULATIONAHA.106.649038

[6] De Luca A, Sarkozy A, Consoli F, Ferese R, Guida V, Dentici ML, et al Familial transposition of the great arteries caused by multiple mutations in laterality genes. Heart. 2010;96(9):673–7. doi: 10.1136/hrt.2009.181685 . 19933292 10.1136/hrt.2009.181685

[7] Goldmuntz E, Bamford R, Karkera JD, dela Cruz J, Roessler E, Muenke M. CFC1 mutations in patients with transposition of the great arteries and double-outlet right ventricle. American journal of human genetics. 2002;70(3):776–80. Epub 2002/01/19. doi: 10.1086/339079 . 11799476 10.1086/339079 PMC384955

[8] Muncke N, Jung C, Rudiger H, Ulmer H, Roeth R, Hubert A, et al Missense mutations and gene interruption in PROSIT240, a novel TRAP240-like gene, in patients with congenital heart defect (transposition of the great arteries). Circulation. 2003;108(23):2843–50. Epub 2003/11/26. doi: 10.1161/01.CIR.0000103684.77636.CD . 14638541 10.1161/01.CIR.0000103684.77636.CD

[9] French VM, van de Laar IM, Wessels MW, Rohe C, Roos-Hesselink JW, Wang G, et al NPHP4 variants are associated with pleiotropic heart malformations. Circulation research. 2012;110(12):1564–74. Epub 2012/05/03. doi: 10.1161/CIRCRESAHA.112.269795 . 22550138 10.1161/CIRCRESAHA.112.269795 PMC3916111

[10] Chhin B, Hatayama M, Bozon D, Ogawa M, Schon P, Tohmonda T, et al Elucidation of penetrance variability of a ZIC3 mutation in a family with complex heart defects and functional analysis of ZIC3 mutations in the first zinc finger domain. Human mutation. 2007;28(6):563–70. Epub 2007/02/14. doi: 10.1002/humu.20480 . 17295247 10.1002/humu.20480

[11] Tan SY, Rosenthal J, Zhao XQ, Francis RJ, Chatterjee B, Sabol SL, et al Heterotaxy and complex structural heart defects in a mutant mouse model of primary ciliary dyskinesia. J Clin Invest. 2007;117(12):3742–52. . 18037990 10.1172/JCI33284 PMC2082149

[12] Aune CN, Chatterjee B, Zhao XQ, Francis R, Bracero L, Yu Q, et al Mouse model of heterotaxy with single ventricle spectrum of cardiac anomalies. Pediatric research. 2008;63(1):9–14. Epub 2007/11/29. doi: 10.1203/PDR.0b013e31815b6926 . 18043505 10.1203/PDR.0b013e31815b6926

[13] Francis RJ, Christopher A, Devine WA, Ostrowski L, Lo C. Congenital heart disease and the specification of left-right asymmetry. American journal of physiology Heart and circulatory physiology. 2012;302(10):H2102–11. Epub 2012/03/13. doi: 10.1152/ajpheart.01118.2011 . 22408017 10.1152/ajpheart.01118.2011 PMC3362104

[14] ATS/ERS recommendations for standardized procedures for the online and offline measurement of exhaled lower respiratory nitric oxide and nasal nitric oxide, 2005. American journal of respiratory and critical care medicine. 2005;171(8):912–30. Epub 2005/04/09. doi: 10.1164/rccm.200406-710ST . 15817806 10.1164/rccm.200406-710ST

[15] Mateos-Corral D, Coombs R, Grasemann H, Ratjen F, Dell SD. Diagnostic value of nasal nitric oxide measured with non-velum closure techniques for children with primary ciliary dyskinesia. The Journal of pediatrics. 2011;159(3):420–4. Epub 2011/04/26. doi: 10.1016/j.jpeds.2011.03.007 . 21514598 10.1016/j.jpeds.2011.03.007

[16] Garrod AS, Zahid M, Tian X, Francis RJ, Khalifa O, Devine W, et al Airway ciliary dysfunction and sinopulmonary symptoms in patients with congenital heart disease. Ann Am Thorac Soc. 2014;11(9):1426–32. doi: 10.1513/AnnalsATS.201405-222OC . 25302410 10.1513/AnnalsATS.201405-222OC

[17] Edelbusch C, Cindric S, Dougherty GW, Loges NT, Olbrich H, Rivlin J, et al Mutation of serine/threonine protein kinase 36 (STK36) causes primary ciliary dyskinesia with a central pair defect. Hum Mutat. 2017;38(8):964–9. doi: 10.1002/humu.23261 . 28543983 10.1002/humu.23261

[18] Kircher M, Witten DM, Jain P, O′Roak BJ, Cooper GM, Shendure J. A general framework for estimating the relative pathogenicity of human genetic variants. Nat Genet. 2014;46(3):310–5. doi: 10.1038/ng.2892 . 24487276 10.1038/ng.2892 PMC3992975

[19] Adzhubei IA, Schmidt S, Peshkin L, Ramensky VE, Gerasimova A, Bork P, et al A method and server for predicting damaging missense mutations. Nature methods. 2010;7(4):248–9. Epub 2010/04/01. doi: 10.1038/nmeth0410-248 . 20354512 10.1038/nmeth0410-248 PMC2855889

[20] Kumar P, Henikoff S, Ng PC. Predicting the effects of coding non-synonymous variants on protein function using the SIFT algorithm. Nature protocols. 2009;4(7):1073–81. Epub 2009/06/30. doi: 10.1038/nprot.2009.86 . 19561590 10.1038/nprot.2009.86

[21] Lek M, Karczewski KJ, Minikel EV, Samocha KE, Banks E, Fennell T, et al Analysis of protein-coding genetic variation in 60,706 humans. Nature. 2016;536(7616):285–91. doi: 10.1038/nature19057 . 27535533 10.1038/nature19057 PMC5018207

[22] Stenson PD, Mort M, Ball EV, Shaw K, Phillips A, Cooper DN. The Human Gene Mutation Database: building a comprehensive mutation repository for clinical and molecular genetics, diagnostic testing and personalized genomic medicine. Hum Genet. 2014;133(1):1–9. doi: 10.1007/s00439-013-1358-4 . 24077912 10.1007/s00439-013-1358-4 PMC3898141

[23] Adams PS, Tian X, Zahid M, Khalifa O, Leatherbury L, Lo CW. Establishing normative nasal nitric oxide values in infants. Respir Med. 2015;109(9):1126–30. doi: 10.1016/j.rmed.2015.07.010 . 26233707 10.1016/j.rmed.2015.07.010

[24] Corbelli R, Bringolf-Isler B, Amacher A, Sasse B, Spycher M, Hammer J. Nasal nitric oxide measurements to screen children for primary ciliary dyskinesia. Chest. 2004;126(4):1054–9. Epub 2004/10/16. doi: 10.1378/chest.126.4.1054 . 15486363 10.1378/chest.126.4.1054

[25] Karadag B, James AJ, Gultekin E, Wilson NM, Bush A. Nasal and lower airway level of nitric oxide in children with primary ciliary dyskinesia. The European respiratory journal: official journal of the European Society for Clinical Respiratory Physiology. 1999;13(6):1402–5. Epub 1999/08/13. . 10445619 10.1183/09031936.99.13614069

[26] Csoma Z, Bush A, Wilson NM, Donnelly L, Balint B, Barnes PJ, et al Nitric oxide metabolites are not reduced in exhaled breath condensate of patients with primary ciliary dyskinesia. Chest. 2003;124(2):633–8. Epub 2003/08/09. . 12907553 10.1378/chest.124.2.633

[27] Eileen Stewart MD PS AD, Xin Tian PhD, Omar Khalifa MD, Peter Wearden MD, PhD, Maliha Zahid MD, PhD, Cecilia W Lo PhD. Airway ciliary dysfunction: Association with adverse postoperative outcomes in non-heterotaxy congenital heart disease patients. The Journal of Thoracic and Cardiovascular Surgery. 2017. Epub 20th September, 2017. https://doi.org/10.1016/j.jtcvs.2017.09.050. 10.1016/j.jtcvs.2017.09.050 PMC6422347 29056267

