# Supplementary material for: Airway ciliary dysfunction and respiratory symptoms in patients with transposition of the great arteries
Source: PLoS One. 2018 Feb 14;13(2):e0191605. doi: 10.1371/journal.pone.0191605 (PMC5812576; doi:10.1371/journal.pone.0191605)
Supplement: S1 Table — (DOCX) [file pone.0191605.s001.docx]

**Table S1. Cilia Motion and nNO in D- and L-TGA Patients**

| **Patient ID** | **TGA Type** | | **Age (yrs)** | | **Sex** | **Race** | **nNO (nl/min)** | **Cilia Motion^*^** | **Cilia Beat Freq (Hz)** |
| --- | --- | --- | --- | --- | --- | --- | --- | --- | --- |
| **Age < 1yr** | |  | |  | | | | | |
| 7422 | D | | 0.003 | | M | Caucasian | 2 | n |  |
| 7306 | D | | 0.003 | | M | Caucasian | 5 | a,g | 8.0 |
| 7381 | D | | 0.003 | | M | Caucasian | 12 | n |  |
| 7097 | D | | 0.01 | | M | Caucasian | - | - |  |
| 7129 | D | | 0.01 | | F | Caucasian | - | n^†‡^ | 4.9 |
| 7151 | D | | 0.01 | | F | Caucasian | - | n^†^ | 3.6 |
| 7295 | D | | 0.01 | | M | Caucasian | 11 | a,c^†‡^ |  |
| 7336 | D | | 0.01 | | M | Caucasian | 20 | n^†‡^ |  |
| 7374 | D | | 0.01 | | F | Caucasian | 13 | n |  |
| 7208 | D | | 0.01 | | M | Caucasian | 9 | a,f | 7.9 |
| 7409 | D | | 0.01 | | F | Caucasian | 10 | a,d^†‡^ |  |
| 7444 | L | | 0.01 | | M | Caucasian | 19 | n |  |
| 7281 | D | | 0.02 | | M | Caucasian | 10 | a,d | 7.8 |
| 7447 | D | | 0.02 | | M | Caucasian | 13 | n^†‡^ |  |
| 7057 | D | | 0.03 | | M | Caucasian | 6 | h |  |
| 7053 | D | | 0.07 | | M | Caucasian | 2 | n | 5.1 |
| 7433 | D | | 0.09 | | M | Caucasian | 12 | n^†‡^ |  |
| 7168 | L | | 0.6 | | F | Caucasian | 10 | n^†‡^ | 5.2 |
| 7004 | D | | 0.7 | | M | Caucasian | 1 | a,b | 5.4 |
| **Age 1-6yrs** |  | |  | |  |  |  |  |  |
| 7002 | D | | 1.3 | | F | Black | 14 | - |  |
| 7273 | D | | 3.0 | | M | Caucasian | 66 | a,b^†‡^ | 6.2 |
| **Age >6 yrs** |  | |  | |  |  |  |  |  |
| 7298 | D | | 9.1 | | M | Caucasian | 248 | a,b | 8.0 |
| 7127 | L | | 9.5 | | M | Caucasian | 574 | a,b | 5.8 |
| 7138 | D | | 10.5 | | F | Caucasian | - | n | 5.3 |
| 7200 | D | | 10.6 | | M | Caucasian | 208 | n | 5.4 |
| 7286 | L | | 10.8 | | M | Caucasian | 287 | a,b,d | 8.3 |
| 7435 | D | | 10.9 | | M | Caucasian | 271 | n^†‡^ |  |
| 7101 | D | | 11.2 | | F | Caucasian | - | b | 6.5 |
| 7013 | D | | 14.1 | | M | Caucasian | 270 | a,b,d |  |
| 7157 | L | | 14.3 | | M | Caucasian | 175 | a,e | 4.5 |
| 7442 | D | | 14.7 | | M | Caucasian | 221 | f |  |
| 7271 | L | | 15.0 | | F | Caucasian | 248 | b,d |  |
| 7269 | D | | 17.3 | | M | Caucasian | 442 | a,b | 5.9 |
| 7216 | L | | 18.1 | | M | Caucasian | 294 | n | 5.1 |
| 7297 | D | | 18.3 | | M | Caucasian | 454 | a,b,i | 5.3 |
| 7367 | D | | 19.8 | | M | Caucasian | 134 | a,b |  |
| 7284 | D | | 19.9 | | M | Caucasian | 177 | n^†‡^ | 6.6 |
| 7148 | D | | 20.2 | | M | Caucasian | 337 | n | 5.9 |
| 7403 | L | | 20.9 | | M | Caucasina | 213 | a,b^†‡^ |  |
| 7120 | L | | 21.6 | | M | Caucasian | 193 | n | 5.5 |
| 7003 | L | | 21.9 | | M | Asian | 117 | - |  |
| 7241 | L | | 22.2 | | F | Caucasian | 282 | a,b,f^†‡^ | 5.4 |
| 7254 | L | | 22.8 | | F | Caucasian | 200 | b,f | 6.2 |
| 7080 | D | | 23.3 | | M | Caucasian | - | n | 3.7 |
| 7244 | D | | 23.7 | | M | Black | 238 | n | 5.2 |
| 7257 | D | | 23.7 | | M | Caucasian | 386 | h |  |
| 7323 | D | | 23.7 | | M | Caucasian | 50 | a,b,i | 5.6 |
| 7233 | L | | 24.6 | | F | Caucasian | 229 | n^†‡^ | 7.7 |
| 7399 | L | | 24.7 | | F | Black | 84 |  |  |
| 7152 | D | | 24.7 | | F | Caucasian | 268 | a,b,f | 5.1 |
| 7089 | D | | 25.0 | | M | Caucasian | - | - |  |
| 7315 | D | | 26.3 | | M | Caucasian | 320 | a,b,g | 5.5 |
| 7235 | D | | 26.6 | | M | Caucasian | 256 | a,b | 7.2 |
| 7115 | D | | 29.0 | | M | Caucasian | - | n^†^ | 5.3 |
| 7172 | D | | 29.0 | | M | Caucasian | 278 | n | 5.7 |
| 7287 | D | | 29.1 | | F | Caucasian | 149 | a,d,e | 4.7 |
| 7320 | L | | 29.8 | | M | Caucasian | 162 | a,b | 5.4 |
| 7248 | D | | 29.9 | | M | Caucasian | 388 | n | 9.1 |
| 7324 | D | | 30.4 | | M | Caucasian | 269 | a,b | 5.6 |
| 7280 | D | | 30.5 | | M | Caucasian | 160 | a,b,d | 5.5 |
| 7069 | D | | 30.9 | | M | Caucasian | - | a,b | 5.6 |
| 7311 | D | | 32.4 | | M | Caucasian | 195 | a,b,g^†‡^ | 7.5 |
| 7110 | D | | 32.9 | | F | Caucasian | - | n | 4.7 |
| 7084 | D | | 34.2 | | M | Caucasian | - | - |  |
| 7188 | D | | 34.6 | | M | Caucasian | 259 | n | 5.8 |
| 7377 | L | | 35.6 | | M | Caucasian | 227 | a,b,f |  |
| 7390 | D | | 36.3 | | F | Caucasian | 272 | b,d |  |
| 7224 | D | | 37.2 | | F | Caucasian | 307 | n | 5.1 |
| 7077 | D | | 42.8 | | F | Caucasian | 281 | n^†‡^ | 5.6 |
| 7096 | D | | 47.0 | | M | Caucasian | - | f | 6.9 |
| 7307 | L | | 47.2 | | F | Caucasian | 168 | a,b | 5.6 |
| 7117 | L | | 47.3 | | F | Caucasian | 171 | n | 9.2 |
| 7261 | L | | 47.8 | | M | Caucasian | 133 | a,b | 5.7 |
| 7118 | L | | 54.4 | | F | Caucasian | 245 | n | 5.1 |

*Cilia Motion Defect: a=asynchronous/dyskinetic, b=incomplete stroke, c=hyperkinetic, d=stiff, e=slow, f=wavy, g= low cilia density, h=cilia aplasia,

i=immotile, n=normal. ^†^Analyzed in reciliated tissue and with ^‡^CM phenotype confirme
