# Supplementary material for: Airway ciliary dysfunction and respiratory symptoms in patients with transposition of the great arteries
Source: PLoS One. 2018 Feb 14;13(2):e0191605. doi: 10.1371/journal.pone.0191605 (PMC5812576; doi:10.1371/journal.pone.0191605)
Supplement: S2 Table — (DOCX) [file pone.0191605.s002.docx]

Table S2. Detailed Cardiovascular Anatomy in TGA Patients^*^

| Patient | Age | Van Praagh | | Atrial Septum | | AV Conn | | Ventric | | Aortic Valve | | |
| --- | --- | --- | --- | --- | --- | --- | --- | --- | --- | --- | --- | --- |
| 7002 | 1.25 | SDD | | ASD | | Bivent | | VSD | | Normal | | |
| 7003 | 21.88 | SLL | |  | | DILV | |  | | Normal | | |
| 7004 | 0.71 | SDD | | ASD | | DILV | | VSD | |  | | |
| 7013 | 14.11 | SDD | | Intact | | Tricuspid atresia | | VSD | |  | | |
| 7053 | 0.07 | SDD | | ASD | | Bivent | | Intact | |  | | |
| 7057 | 0.03 | SDD | | ASD | |  | | Intact | |  | | |
| 7069 | 30.87 | SDD | | Intact | |  | | Intact | | Normal | | |
| 7077 | 42.75 | SDD | |  | | Bivent | | Intact | | Normal | | |
| 7080 | 23.32 | SDD | | Intact | | Bivent | | VSD | | Normal | | |
| 7084 | 34.18 | SDD | |  | |  | |  | | Normal | | |
| 7089 | 24.99 | SDD | | ASD | | Tricuspid atresia | | mVSD | | Normal | | |
| 7096 | 47.03 | SDD | |  | | Bivent | |  | |  | | |
| 7097 | 0.01 | SDD | | ASD | |  | | Intact | |  | | |
| 7101 | 11.16 | SDD | |  | | DILV | |  | |  | | |
| 7110 | 0.1 | SDD | |  | | Bivent | | Intact | | Normal | | |
| 7115 | 28.94 | SDD | |  | | Bivent | | VSD | | Normal | | |
| 7117 | 47.22 | SLL | |  | | Bivent-DSC | | Intact | |  | | |
| 7118 | 54.34 | SLL | | Intact | | Bivent-DSC | | VSD | | Normal | | |
| 7120 | 21.58 | SLL | |  | | Bivent-DSC | | Intact | | Normal | | |
| 7127 | 9.53 | SLL | |  | | DILV | | VSD | |  | | |
| 7129 | 0.01 | SDD | | Intact | | Bivent | | mVSD | | Normal | | |
| 7138 | 10.53 | SDD | |  | | Bivent | | VSD | | Normal | | |
| 7148 | 20.21 | SDD | | Intact | | Bivent | | VSD | | Normal | | |
| 7151 | 0.01 | SDD | | Intact | | Bivent | | Intact | | Normal | | |
| 7152 | 24.68 | SDD | | ASD | | Bivent | | Intact | |  | | |
| 7157 | 14.26 | SLL | | Intact | | Bivent-DSC | | Intact | | Normal | | |
| 7168 | 0.63 | SLL | |  | | DILV | | VSD | | Stenotic | | |
| 7172 | 28.94 | SDD | | Intact | | Bivent | | VSD | |  | | |
| 7188 | 34.59 | SDD | | Intact | | Bivent | |  | |  | | |
| 7200 | 10.56 | SDD | |  | | Bivent | | VSD | | Normal | | |
| 7208 | 0.01 | SDD | |  | |  | | Intact | |  | | |
| 7216 | 18.11 | SLL | | Intact | | Bivent-DSC | | Intact | | Normal | | |
| 7224 | 37.12 | SDD | |  | | Tricuspid atresia | | VSD | |  | | |
| 7233 | 24.57 | SLL | |  | | Bivent-DSC | | Intact | | Normal | | |
| 7235 | 26.54 | SDD | | Intact | | Bivent | | Intact | | Normal | | |
| 7241 | 22.15 | SLL | | ASD | | Bivent-DSC | | VSD | |  | | |
| 7244 | 23.67 | SDD | | ASD | | Tricuspid atresia | | VSD | | Normal | | |
| 7248 | 29.83 | SDD | |  | | Bivent | | Intact | |  | | |
| 7254 | 22.78 | SLL | | Intact | | Bivent-DSC | | VSD | | Normal | | |
| 7257 | 23.72 | SDD | |  | | Tricuspid atresia | | mVSD | | Normal | | |
| 7261 | 47.79 | SLL | | ASD | | Biven-DSC | | VSD | | Normal | | |
| 7269 | 17.28 | SDD | |  | | Bivent | | Intact | | Normal | | |
| 7271 | 15.04 | SLL | |  | | DILV | | VSD | |  | | |
| 7273 | 0.02 | SDD | | Intact | | Bivent | | Intact | | Normal | | |
| 7280 | 30.53 | SDD | |  | | Bivent | |  | |  | | |
| 7281 | 0.02 | SDD | |  | | Bivent | | VSD | |  | | |
| 7284 | 19.91 | SDD | | Intact | | Bivent | | Intact | | Normal | | |
| 7286 | 10.83 | SLL | | ASD | | Bivent-DSC | | Intact | |  | | |
| 7287 | 29.06 | SDD | | Intact | | Bivent | | Intact | | Normal | | |
| 7295 | 0.01 | SDD | | intact | | Bivent | | Intact | | Normal | | |
| 7297 | 18.33 | SDD | | Intact | | Bivent | | Intact | | Normal | | |
| 7298 | 9.07 | SDD | |  | | Bivent | | Intact | |  | | |
| 7306 | 0.00 | SDD | | ASD | | Bivent | | Intact | |  | | |
| 7307 | 47.17 | SLL | |  | | Bivent-DSC | | Intact | |  | | |
| 7311 | 32.43 | SDD | | intact | | Bivent | | VSD | | Normal | | |
| 7315 | 26.34 | SDD | |  | | Bivent | | Intact | | Normal | | |
| 7320 | 29.81 | SLL | | intact | | Bivent-DSC | | Intact | | Normal | | |
| 7323 | 23.72 | SDD | | Intact | | Bivent | | Intact | |  | | |
| 7324 | 30.38 | SDD |  | | Bivent | | Intact | |  | | |  |
| 7336 | 0.01 | SDD | intact | | Bivent | | Intact | | Normal | | |  |
| 7364 | 23.51 | SLL |  | | Bivent-DSC | | mVSD | | Normal | | |  |
| 7367 | 19.83 | SDD | Intact | | Bivent | | VSD | | Normal | | |  |
| 7374 | 0.01 | SDL |  | | Tricuspid atresia | | mVSD | | Normal | | |  |
| 7377 | 35.6 | SLL | Intact | | Bivent-DSC | | Intact | |  | | |  |
| 7381 | 0.003 | SDD | Intact | | Bivent | |  | | | |  |  |
| 7390 | 36.32 | SDD |  | | Bivent | | Intact | | | |  |  |
| 7399 | 24.67 | SLL |  | | Bivent-DSC | | VSD | | | |  |  |
| 7403 | 20.86 | SLL | Intact | | Bivent-DSC | | Intact | | | | Normal |  |
| 7409 | 0.01 | SDD | ASD | | Tricuspid atresia | | mVSD | | | | Normal |  |
| 7422 | 0.003 | SDD | Intact | | Bivent | | Intact | | | | Normal |  |
| 7433 | 0.09 | SDD | Intact | | Bivent | | VSDs | | | | Normal |  |
| 7435 | 10.92 | SDD |  | | Bivent | |  | | | |  |  |
| 7442 | 14.73 | SDD | Intact | | Bivent | | Intact | | | | Normala |  |
| 7444 | 0.01 | SLL | Intact | | Univent-DILV | | VSD | |  | | |  |
| 7447 | 0.02 | SDD | ASD | | Univent-DILV | | VSD | | Normal | | |  |

Yellow high-lighted patients had whole exome sequencing performed. ASD: atrial septal defect; Bivent: biventricular; Bivent-DSC: biventricular discordance, DILV: double inlet left ventricle, Hypo: hypoplastic, Univent-AbR: univentricular with abnormal right; Ebstein’s: Ebstein’s anomaly, mVSD: muscular ventricular septal defect, SAo: straddling aortic; Sm/Str/Cleft: small straddling cleft.
