# Supplementary material for: Airway ciliary dysfunction and respiratory symptoms in patients with transposition of the great arteries
Source: PLoS One. 2018 Feb 14;13(2):e0191605. doi: 10.1371/journal.pone.0191605 (PMC5812576; doi:10.1371/journal.pone.0191605)
Supplement: S3 Table — (DOCX) [file pone.0191605.s003.docx]

Table S3. Nasal NO values by age groups in PCD and healthy Controls[^5^](#_ENREF_5).

|  | Total N | Age in yrs  (Mean±SD) | nNO in nl/min  (Mean±SD) |
| --- | --- | --- | --- |
| Controls  <1 yr | 8 | 0.50 ± 0.27 | 68.81 ± 23.36 |
| Controls  1-6 yrs | 82 | 2.94 ± 1.39 | 123.31 ± 59.58 |
| Controls  >6 yrs | 26 | 32.01 ± 9.28 | 316.32 ± 90.03 |
| PCD  <1 yr | 6 | 0.24 ± 0.13 | 7.78 ± 5.18 |
| PCD  1-6 yrs | 17 | 3.3 ± 1.68 | 19.65 ± 13.68 |
| PCD  >6 yrs | 18 | 19.56 ± 16.73 | 16.46 ± 10.52 |
