# Supplementary material for: Airway ciliary dysfunction and respiratory symptoms in patients with transposition of the great arteries
Source: PLoS One. 2018 Feb 14;13(2):e0191605. doi: 10.1371/journal.pone.0191605 (PMC5812576; doi:10.1371/journal.pone.0191605)
Supplement: S4 Table — (DOCX) [file pone.0191605.s004.docx]

| Patient ID  Table S4. Respiratory Symptoms in Patients >6yrs of Age | TGA Type | Ciliary Motion | Otitis Media | Sinusitis | Chronic Nasal Congestion | Chronic Cough | Chronic Sputum | Neonatal Pneumonia RDS | Bronchitis | Pneumonia, Chest Infections |
| --- | --- | --- | --- | --- | --- | --- | --- | --- | --- | --- |
| 7297 | D-TGA | Abnormal | Yes | No | No | No | No | No | No | No |
| 7152 | D-TGA | Abnormal | No | No | No | No | No | No | No | No |
| 7287 | D-TGA | Abnormal | Yes | Yes | Yes | No | No | No | No | No |
| 7013 | D-TGA | Abnormal | No | No | No | No | No | No | No | No |
| 7367 | D-TGA | Abnormal | Yes | Yes | Yes | No | No | No | No | Yes |
| 7069 | D-TGA | Abnormal | No | No | No | No | No | No | No | No |
| 7311 | D-TGA | Abnormal | No | No | No | No | No | Yes | No | Yes |
| 7323 | D-TGA | Abnormal | No | No | No | Yes | No | No | No | Yes |
| 7390 | D-TGA | Abnormal | Yes | Yes | No | No | No | No | No | No |
| 7269 | D-TGA | Abnormal | Yes | Yes | No | No | No | No | Yes | Yes |
| 7096 | D-TGA | Abnormal | Yes | Yes | Yes | Yes | Yes | No | Yes | Yes |
| 7324 | D-TGA | Abnormal | No | Yes | No | No | No | No | No | No |
| 7298 | D-TGA | Abnormal | No | Yes | No | Yes | No | Yes | Yes | Yes |
| 7235 | D-TGA | Abnormal | Yes | No | No | No | No | No | No | No |
| 7280 | D-TGA | Abnormal | No | Yes | Yes | No | No | No | No | No |
| 7442 | D-TGA | Abnormal | Yes | No | No | No | No | No | No | No |
| 7101 | D-TGA | Abnormal | No | No | No | No | No | No | No | No |
| 7315 | D-TGA | Abnormal | No | No | Yes | No | No | No | No | No |
| 7271 | L-TGA | Abnormal | No | No | No | No | No | No | No | No |
| 7403 | L-TGA | Abnormal | No | No | No | No | No | No | No | No |
| 7261 | L-TGA | Abnormal | No | No | Yes | No | No | No | No | No |
| 7377 | L-TGA | Abnormal | No | No | No | No | No | No | No | No |
| 7127 | L-TGA | Abnormal | No | No | No | No | No | No | No | No |
| 7307 | L-TGA | Abnormal | No | Yes | Yes | Yes | No | No | No | No |
| 7320 | L-TGA | Abnormal | Yes | No | No | No | No | No | Yes | No |
| 7364 | L-TGA | Abnormal | No | No | No | No | No | No | No | No |
| 7254 | L-TGA | Abnormal | No | No | No | No | No | No | No | No |
| 7157 | L-TGA | Abnormal | Yes | Yes | No | No | No | Yes | No | Yes |
| 7286 | L-TGA | Abnormal | Yes | No | No | No | No | No | Yes | No |
| 7241 | L-TGA | Abnormal | Yes | Yes | No | No | No | No | No | Yes |
| 7399 | L-TGA | Abnormal | No | Yes | No | No | No | No | No | No |
| 7244 | D-TGA | Normal | No | No | No | No | No | No | No | No |
| 7110 | D-TGA | Normal | Yes | No | No | No | No | No | No | No |
| 7148 | D-TGA | Normal | Yes | No | No | No | No | No | No | No |
| 7077 | D-TGA | Normal | No | No | No | No | No | No | No | No |
| 7200 | D-TGA | Normal | No | No | No | No | No | No | No | No |
| 7080 | D-TGA | Normal | No | No | No | No | No | No | No | No |
| 7115 | D-TGA | Normal | No | No | No | No | No | No | No | No |
| 7172 | D-TGA | Normal | Yes | No |  | No | No | Yes | No |  |
| 7188 | D-TGA | Normal | No | No | No | No | No | No | No | No |
| 7284 | D-TGA | Normal | No | No | No | No | No | No | No | No |
| 7435 | D-TGA | Normal | No | No | No | No | No | No | No | No |
| 7138 | D-TGA | Normal | No | No | No | No | No | No | No | No |
| 7248 | D-TGA | Normal | No | No | No | No | No | No | No | No |
| 7233 | L-TGA | Normal | Yes | No | Yes | Yes | Yes | No | Yes | No |
| 7120 | L-TGA | Normal | No | No | No | No | No | No | No | No |
| 7118 | L-TGA | Normal | No | No | No | No | No | No | No | No |
| 7117 | L-TGA | Normal | No | No | No | No | No | No | No | No |
| 7216 | L-TGA | Normal | Yes | No | No | No | No | No | No | No |
