# Supplementary material for: Airway ciliary dysfunction and respiratory symptoms in patients with transposition of the great arteries
Source: PLoS One. 2018 Feb 14;13(2):e0191605. doi: 10.1371/journal.pone.0191605 (PMC5812576; doi:10.1371/journal.pone.0191605)
Supplement: S1 Fig — (DOCX) [file pone.0191605.s005.docx]

Figure S1: Cilia Motion Analysis and nNO Measurement in the TGA Cohort.

**TGA Cohort=75**

Cilia Motion

nNO

5

58

9

2

44

14

3

4
