# Supplementary material for: Airway ciliary dysfunction and respiratory symptoms in patients with transposition of the great arteries
Source: PLoS One. 2018 Feb 14;13(2):e0191605. doi: 10.1371/journal.pone.0191605 (PMC5812576; doi:10.1371/journal.pone.0191605)
Supplement: S1 Methods — (DOC) [file pone.0191605.s013.doc]

Supplemental Methods

Patient Recruitment

The study protocol was approved by the University of Pittsburgh institutional board review. Informed consent was obtained from all patients and from parents/guardians in case of minors. We recruited 75 patients with TGA, 51 (68%) of whom were male, with 54 (72%) having D-TGA and 21 (28%) with L-TGA (Figure 1). Mean age was 18.6 yrs (±14.7), with 19 < 1 yr of age, 2 were 1 - 6 yrs, and 54 were >6 yrs of age. The majority of patients (71; 95%) were Caucasian, and the remainder African American (3), and Asian Indian (1). We excluded all patients with any laterality disturbance in any organ in the body [^1^](#_ENREF_1) (Supplemental Table S1 and Table S2). The recruitment protocol entailed obtaining consent, administration of a family and medical history questionnaire to the patient or parents of the patient. This included gathering specific data on respiratory symptoms (Supplementary Table S3). We also obtained a blood draw for DNA, and the assessment of nNO and procurement of a nasal scrape for respiratory ciliary motion analysis.

Nasal nNO Measurement

Given nNO measurements can be affected by acute upper or lower respiratory measurements, every effort was made to sample the patient at their steady baseline when they were free of any symptoms of upper or lower respiratory tract infection. In case of abnormally high or low values, repeat measurements were obtained whenever possible. Nasal nitric oxide (nNO) measurements were made by inserting an NO inert line into one nostril while the contralateral nostril was left open. For measurement purposes, a chemiluminescence nitric oxide analyzer (CLD 88SP, ECO PHYSICS AG, Duerten, Switzerland) was utilized with a constant sample flow rate of 0.3 liters/min. nNO production was calculated as the product of measured nNO concentration in parts per billion (ppb) and the sample flow rate (L/min). For participants under 6 years of age, nNO was measured using the tidal breath sampling method. For participants over 6 years of age, resistor measurements were made using the velum closure technique whereby participant exhaled forcibly against resistance for 20-40 seconds. All measurements were made in triplicate (tidal breathing) or quadruplicate (velum-closure technique), and average values were used in statistical analyses.

Culturing and Reciliation of Patient Derived Nasal Epithelia

The tissue was first placed in culture on rat-tail collagen coated plates in media containing Ultroser G (Pall Life Sciences) which causes deciliation and proliferation of the respiratory epithelia. After reaching confluence, the tissue was then removed with collagenase and placed in suspension culture on an orbital shaker in medium containing Nuserum (Becton Dickson). Once in suspension, the cells aggregate, form stable spheroids and undergo reciliation, from which ciliary motion was recorded by video-microscopy and analyzed in the same manner as the original nasal scrape to determine the CM phenotype. Investigators analyzing the initial nasal scrape as well as the reciliated tissue ciliary motion were blinded to the patient’s phenotype as well as nNO levels.

Whole Exome Sequencing

Whole-exome sequencing analysis was performed using Agilent SureSelect All Exon Kit V4 and Illumina High-seq2000 sequencer. An average coverage of ~100x was achieved for each exome. Sequence data were analyzed using GATK pipeline (http://www.broadinstitute.org/gsa) and sequence variants were further annotated using annovar (**www.openbioinformatics.org/annovar**) and custom scripts. 30 known PCD genes ([**http://ghr.nlm.nih.gov/condition/primary-ciliary-dyskinesia/show/Related+Gene%28s%29**](http://ghr.nlm.nih.gov/condition/primary-ciliary-dyskinesia/show/Related+Gene(s))) were examined for novel or rare coding variants (nonsynonymous, frameshift, splicing), defined as those with <0.8% allele frequency in the 1000 Genomes ([www.1000genomes.org](http://www.1000genomes.org/)) NHLBI exome (evs.gs.washington.edu/) databases. Pathogenicity of these rare/novel PCD gene coding mutations were assessed by PolyPhen-2 [^2^](#_ENREF_2), SIFT [^3^](#_ENREF_3) and CADD Score algorithms [^4^](#_ENREF_4).

Supplemental Reference
